# HO-1 Up-regulation and PINK1/Parkin-Mediated Mitophagy Contribute to the Anti-tumor Effects of Metochalcone in Colorectal Cancer

**DOI:** 10.34133/research.1387

**Published:** 2026-08-04

**Authors:** Pingting Chen, Junsha An, Mingyu Han, Yuhan Wang, Xue Li, Jianning Yang, Heng Zhang, Huali Fan, Zhaokai Zhou, Hailin Tang, Fu Peng

**Affiliations:** ^1^ West China School of Pharmacy, Sichuan University, Chengdu 610041, China.; ^2^ Department of Urology, The Second Xiangya Hospital of Central South University, Changsha, Hunan 410011, China.; ^3^ State Key Laboratory of Oncology in South China, Guangdong Provincial Clinical Research Center for Cancer, Sun Yat-Sen University Cancer Center, Guangzhou 510060, China.; ^4^ Key Laboratory of Drug-Targeting and Drug Delivery System of the Education Ministry, Sichuan Engineering Laboratory for Plant-Sourced Drug and Sichuan Research Center for Drug Precision Industrial Technology, Sichuan University, Chengdu, 610041, China.

## Abstract

Colorectal cancer (CRC) remains a global health challenge with rising incidence in younger populations and limited efficacy of current chemotherapies. In this study, we investigated the anti-tumor mechanisms of metochalcone (MET), a natural chalcone derivative, in CRC. Using a combination of transcriptomic, proteomic, and targeted in vitro and in vivo assays, we found that MET substantially reduced viability, migration, and invasion and arrested the cell cycle at the G_0_/G_1_ phase in HCT-116 CRC cells. Integrative analysis identified heme oxygenase-1 (HO-1) as a key target, and molecular docking and cellular thermal shift assay confirmed that MET directly binds to and stabilizes the HO-1 protein. Mechanistically, MET treatment led to mitochondrial dysfunction, characterized by increased reactive oxygen species and decreased membrane potential, which in turn activated PTEN induced kinase 1 (PINK1)/Parkin-mediated mitophagy. In 2 distinct mouse models of CRC, MET potently suppressed tumor growth. Furthermore, 16S ribosomal RNA gene sequencing revealed that MET treatment was associated with changes in gut microbiota composition in mice, including an increased relative abundance of beneficial *Lactobacillus* and a decreased abundance of pro-inflammatory *Desulfovibrionaceae*. Our findings demonstrate that MET exerts multifaceted anti-tumor effects, including direct targeting of HO-1 and activation of PINK1/Parkin-mediated mitophagy, accompanied by alterations in gut microbiota composition.

## Introduction

Colorectal cancer (CRC) remains a major global malignancy. Approximately 1.9 million new cases and 904,000 deaths occur annually, ranking CRC the third most commonly diagnosed cancer and the second leading cause of cancer-related mortality [1]. While incidence and mortality rates in older populations in developed nations have seen a decline due to improved screening and treatment, a concerning trend has emerged: the incidence of early-onset CRC in individuals under 55 has been rising annually by 1% to 2% since the mid-1990s [2,3]. The etiological factors and pathogenetic mechanisms of CRC development are complex and heterogeneous [4]. Therefore, CRC has different treatments and a complex classification. Recent evidence indicates that dysregulation of intracellular sodium can influence cancer metabolism, immune responses, and therapeutic resistance [5].

In general, the standard management for CRC is early surgical resection combined with radiotherapy, immunotherapy, and targeted therapy [6]. Current chemotherapy includes various cytotoxic drugs such as 5-fluorouracil (5-FU), oxaliplatin (OX), capecitabine (CAPE), and irinotecan (IRI), administered as monotherapy or in combination regimens such as FOLFOX, CAPEOX, FOLFIRI, and FOLFIRINOX [7]. Considerable research has been dedicated to unraveling the mechanisms behind chemotherapy resistance, particularly 5-FU resistance. For instance, a recent study identified a novel target, the tsRNA-GlyGCC-mediated pathway, which promotes 5-FU resistance by regulating the transcription factor SPIB [8]. Nevertheless, chemotherapy still dominates the treatment of CRC, but it is limited by systemic toxicity, acquired resistance, and low tumor-specific selectivity [9].

Mitophagy, a selective form of autophagy, is crucial for maintaining cellular homeostasis by eliminating damaged or dysfunctional mitochondria [10]. This essential process has garnered significant attention in cancer biology, where its role is increasingly recognized as a double-edged sword [11]. On one hand, mitophagy can promote tumor cell survival and proliferation by clearing unhealthy mitochondria, thereby reducing reactive oxygen species (ROS) and metabolic stress, and contributing to therapeutic resistance [12]. Conversely, it can also act as a tumor suppressor by preventing the accumulation of dysfunctional mitochondria, which might otherwise fuel oncogenic signaling or genomic instability [13]. Excessive mitophagy may lead to a significant reduction in the number of mitochondria within tumor cells and a decline in their function, ultimately resulting in cell death [14]. Dysregulation of mitophagy leads to the accumulation of defective mitochondria and has been implicated in the initiation and progression of cancer [15–17].

The gut microbiota and host metabolism have emerged as critical regulators of CRC biology. As a major component of the tumor microenvironment, gut microbes influence CRC initiation, progression, and therapy response through immune modulation and metabolic cross talk [18–20], with recent evidence highlighting that harnessing the intratumoral microbiome and associated immune responses can markedly potentiate therapeutic outcomes [21].

Natural products and their derivatives have long served as a rich source of anticancer leads because of their structural diversity and multitarget properties. Indeed, many successful anticancer drugs, such as paclitaxel (derived from *Taxus baccata*), vincristine (derived from *Catharanthus roseus*), and camptothecin (derived from *Camptotheca acuminata*), are directly derived from natural compounds, underscoring their immense therapeutic potential. Among them, chalcone scaffolds have shown promising antiproliferative and pro-apoptotic activities in various cancers, acting on multiple signaling pathways and cellular processes [22–24]. This multitargeted efficacy is highly sought after, particularly given the heterogeneous spectrum of mechanisms underlying CRC, which involves not only cellular proliferation but also chronic inflammation and compromised intestinal barrier function. Indeed, natural derivatives are continually being explored for their potential to modulate these critical upstream pathways in CRC pathogenesis, such as restoring intestinal integrity and suppressing inflammation [25,26]. Recent work demonstrated that a chalcone compound, isoliquiritigenin (ISL), suppresses colorectal tumor progression via inhibition of the FGFR4–FASN axis that governs lipid metabolism [27]. Building upon the established anticancer properties of chalcones, including ISL, metochalcone (MET), a well-characterized derivative, has previously shown significant antiproliferative effects in various carcinoma models [28,29]. Specifically, it inhibits cell proliferation, induces cell-cycle arrest, and reduces tumor growth in breast and lung cancer cells by modulating pathways like JAK2/STAT3 and p53. This existing evidence of its potent anti-tumor activity in other solid malignancies, coupled with its structural similarity to ISL, provided a strong rationale for its investigation in CRC. Therefore, MET, a chalcone derivative, emerged from our preliminary screens as a candidate compound with anti-CRC activity. Here, we combined integrative transcriptomic/proteomic analyses with targeted in vitro and in vivo experiments to characterize the anti-tumor effects of MET and to elucidate its potential mechanisms of action, focusing on mitophagy and gut microbiota modulation.

## Results

### MET inhibits CRC cell growth, cycle progression, and motility

**Fig. 1.**
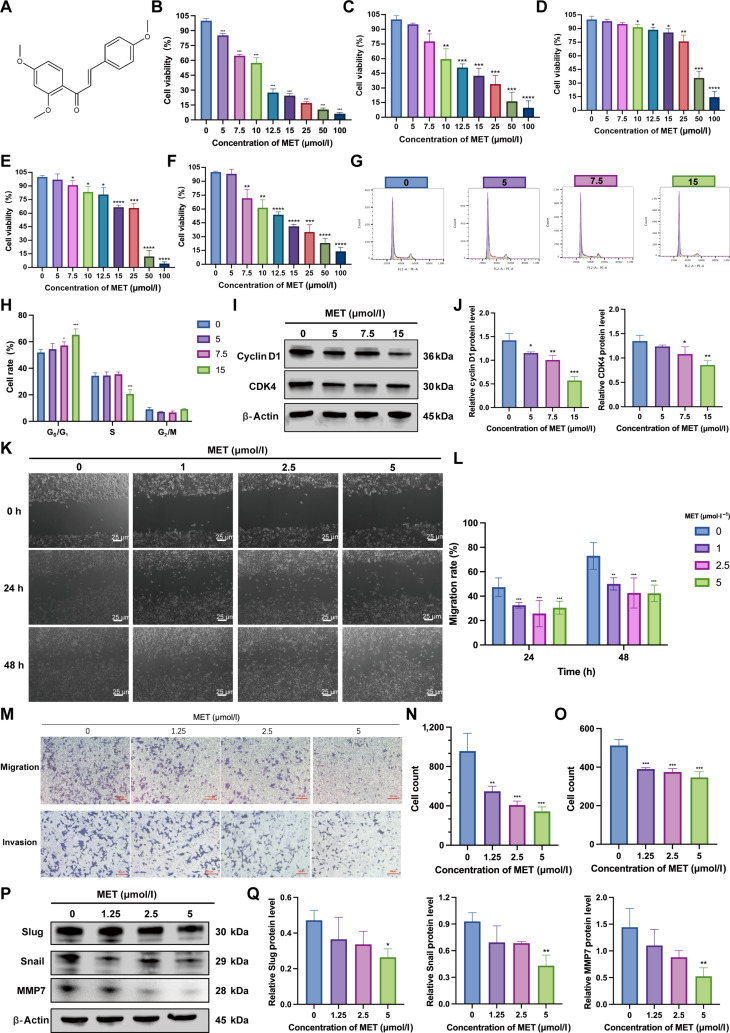
Metochalcone (MET) inhibits colorectal cancer (CRC) cell proliferation, induces G_0_/G_1_ phase cell-cycle arrest, and suppresses the migration and invasion of CRC cells by inhibiting the epithelial–mesenchymal transition (EMT) process. (A) Chemical structure of MET. (B to F) Viability of CRC cells (HCT-116, HT-29, MC38, and SW480 cells) and normal colonic epithelial cells (NCM460 cells) treated with MET (0 to 100 μM) for 24 h, assessed by the Cell Counting Kit-8 (CCK-8) assay. (G and H) Cell-cycle distribution of HCT-116 cells after treatment with MET (5, 7.5, and 15 μM) for 24 h. (I and J) Western blot analysis of cell-cycle regulatory proteins, cyclin D1 and CDK4, in HCT-116 cells after MET treatment. (K and L) Representative images and statistical analysis of wound-healing assays showing the migratory ability of HCT-116 cells treated with MET (5 and 15 μM). The migration rate was measured at 0 and 24 h. (M to O) Representative images and quantitative analysis of transwell assays showing the migratory and invasive ability of HCT-116 cells after MET treatment. (P and Q) The expression levels of EMT-related proteins, MMP7, Snail, and Slug, in MET-treated cells.

MET (structure shown in Fig. 1A) demonstrated inhibitory effects on various CRC cell lines, showing the best efficacy in HCT-116 cells with an IC_50_ of 11.61 μM (Fig. 1B). In comparison, the IC_50_ values for MET in other CRC cell lines were, sequentially, 14.42 μM for HT-29 cells, 25.92 μM for SW480 cells, and 39.22 μM for MC38 cells (Fig. 1C to E). Furthermore, MET exhibited lower toxicity to human normal colonic epithelial cells (NCM460) compared to its cytotoxic effects on HCT-116 cells across all tested concentrations (Fig. 1F). Flow cytometric cell-cycle analysis (Fig. 1G and H) demonstrated that MET (5, 7.5, and 15 μM) increased the G_0_/G_1_ population from 51.9% ± 2.3% in controls to 54.4% ± 4.2%, 57.2% ± 2.7%, and 65.3% ± 4.4%, respectively (*P* < 0.05), indicating G_0_/G_1_ arrest. Western blot (Fig. 1I and J) confirmed the down-regulation of cyclin D1 and CDK4 upon MET treatment, corroborating the blockade of G_1_/S transition. Wound-healing and transwell assays (Fig. 1K to O) showed that MET reduced migration and invasion (*P* < 0.01). Additionally, MET decreased the expression of epithelial–mesenchymal transition markers MMP7, Snail, and Slug (Fig. 1P and Q), further supporting its inhibitory effect on CRC cell motility.

### Transcriptomic and proteomic profiling identify MET-driven PINK1/Parkin-associated mitophagy

**Fig. 2.**
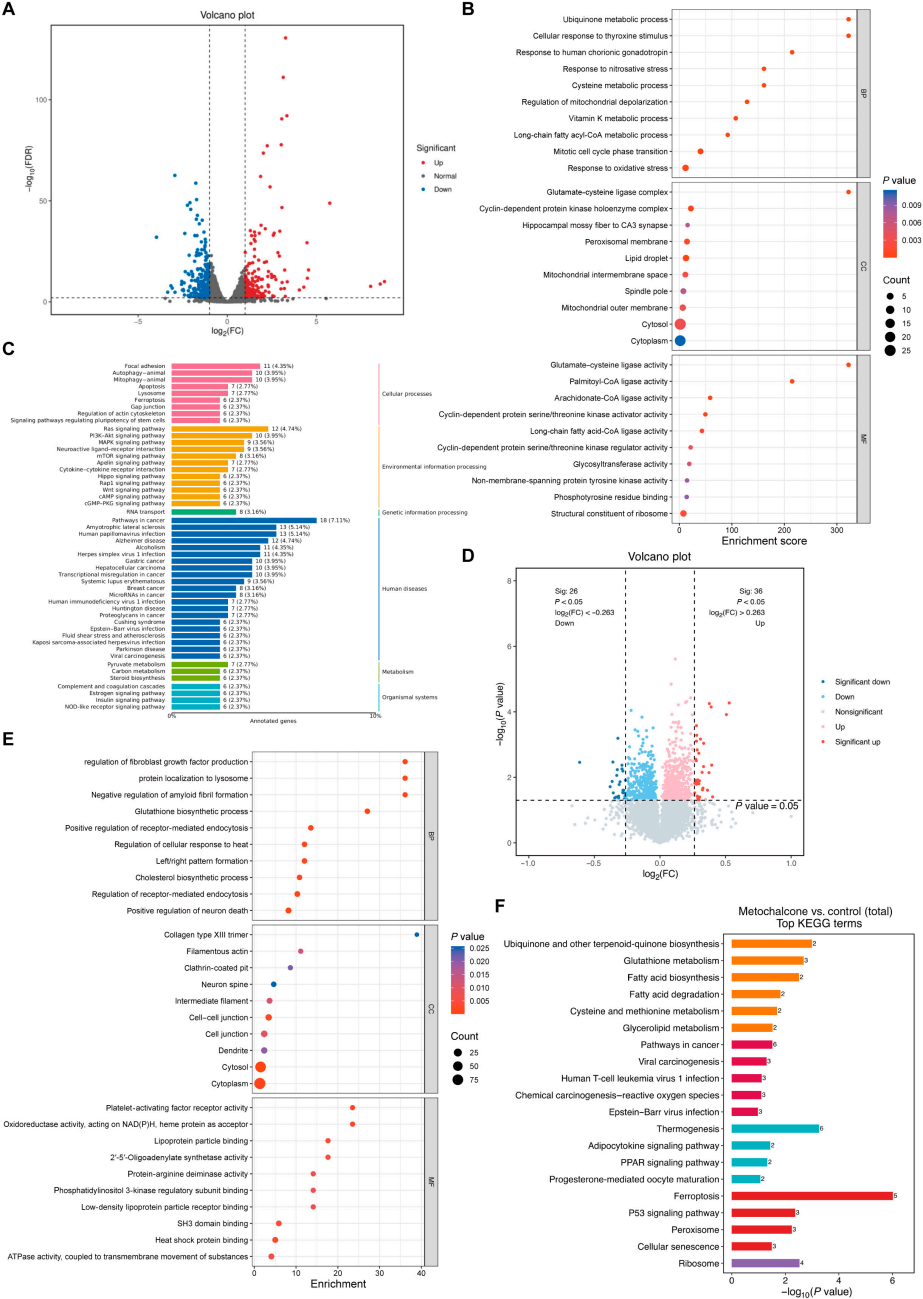
Transcriptomic analysis and proteomic profiling of metochalcone (MET)-treated HCT-116 cells. (A to C) Differential gene expression and functional enrichment analyses, including Gene Ontology (GO) and Kyoto Encyclopedia of Genes and Genomes (KEGG) pathways, following MET treatment. (D to F) Differential proteomic profiling and associated GO and KEGG enrichment analyses in MET-treated cells.

RNA sequencing analysis identified 604 differentially expressed genes (DEGs) in MET-treated HCT-116 cells, of which 319 were up-regulated and 285 down-regulated (Fig. 2A). Gene Ontology (GO) enrichment of these DEGs revealed significant association with the glutathione biosynthetic process, protein localization to lysosomes, and oxidoreductase activity (Fig. 2B), while Kyoto Encyclopedia of Genes and Genomes (KEGG) pathway analysis highlighted mitophagy, autophagy, and ferroptosis (Fig. 2C).

Proteomic profiling identified 62 differentially expressed proteins (DEPs), including 36 up-regulated and 26 down-regulated (Fig. 2D). GO terms enriched among DEPs included regulation of mitochondrial depolarization and the ubiquinone metabolic process (Fig. 2E), and KEGG pathways featured peroxisome, p53 signaling, ferroptosis, and glutathione metabolism (Fig. 2F).

**Fig. 3.**
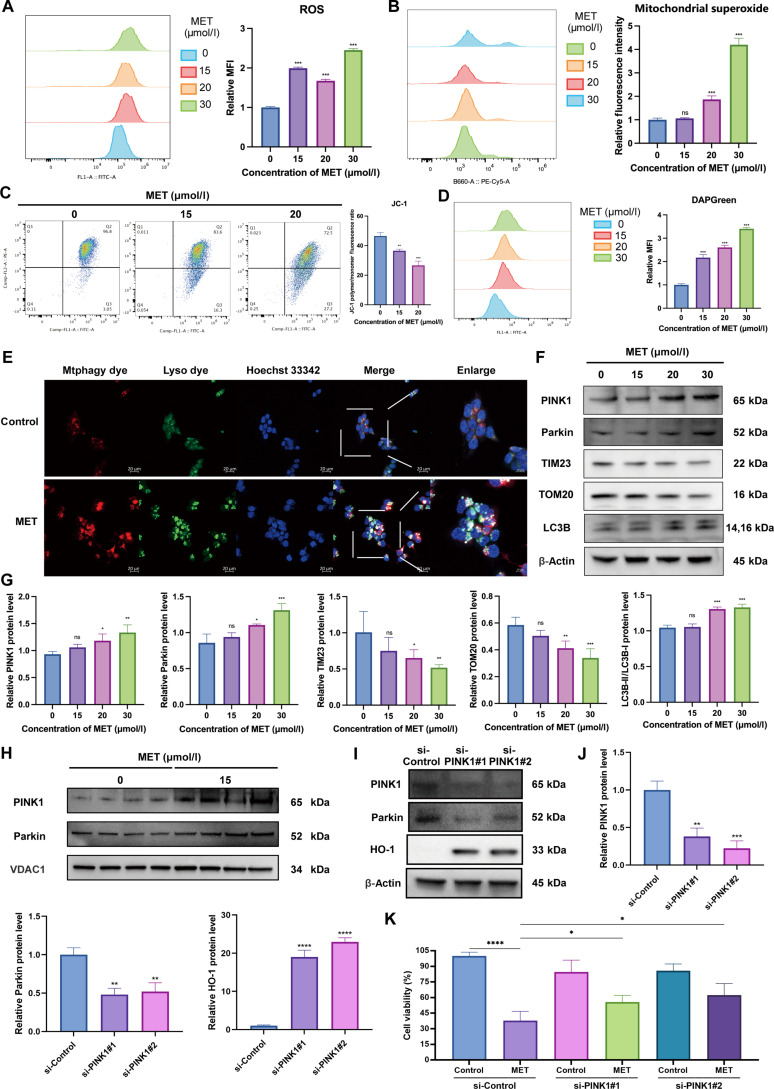
Metochalcone (MET) mediates mitophagy by regulating the PTEN induced kinase 1 (PINK1)/Parkin pathway. (A) Quantitative analysis of intracellular reactive oxygen species (ROS) levels in HCT-116 cells treated with MET. (B) Quantitative analysis of mitochondrial superoxide levels. (C) JC-1 staining for the assessment of ΔΨm. (D) Detection of autophagy activity by DAPGreen upon MET treatment. (E) Representative images and quantitative analysis of costaining with Mtphagy and Lyso dyes. (F to H) The expression of the mitochondrial markers TOM20 and TIM23 and the mitophagy-related proteins LC3B-II, PINK1, and Parkin in whole-cell lysates and purified mitochondrial fractions after MET treatment. (I and J) The expression of PINK1, Parkin, and heme oxygenase-1 (HO-1) protein in HCT-116 cells after si-PINK1 knockdown. (K) Cell viability of HCT-116 cells treated with MET (20 μM) following si-PINK1 knockdown.

Omics analysis suggested that MET might induce autophagy, particularly mitophagy, in CRC cells. We therefore investigated relevant markers. MET markedly increased intracellular ROS (Fig. 3A) and mitochondrial superoxide (Fig. 3B) and decreased mitochondrial membrane potential (ΔΨm) in a dose-dependent manner (Fig. 3C). Moreover, the DAPGreen fluorescence intensity rose significantly (Fig. 3D), reflecting an accumulation of autophagosomes, and costaining with the Mtphagy and Lyso dyes showed enhanced overlap (Fig. 3E), confirming that MET induced mitophagy in CRC cells.

The PTEN induced kinase 1 (PINK1)/Parkin pathway is intimately involved in mitophagy. Western blot analysis showed the down-regulation of the mitochondrial markers TOM20 and TIM23 and the up-regulation of LC3B-II, PINK1, and Parkin in whole-cell and purified mitochondrial fractions (Fig. 3F to H). These data demonstrated that MET activated PINK1/Parkin-mediated mitophagy, leading to CRC cell death.

Subsequently, we knocked down PINK1 expression using small interfering RNA (siRNA) and observed a concomitant down-regulation of Parkin, while heme oxygenase-1 (HO-1) expression was significantly up-regulated (Fig. 3I and J). Importantly, PINK1 knockdown partially rescued MET’s inhibitory effect on HCT-116 cells, indicating that PINK1/Parkin-mediated mitophagy contributes to MET’s cytotoxic effects (Fig. 3K).

### HO-1 acts as a direct target of MET and participates in MET-mediated inhibition of CRC cells

**Fig. 4.**
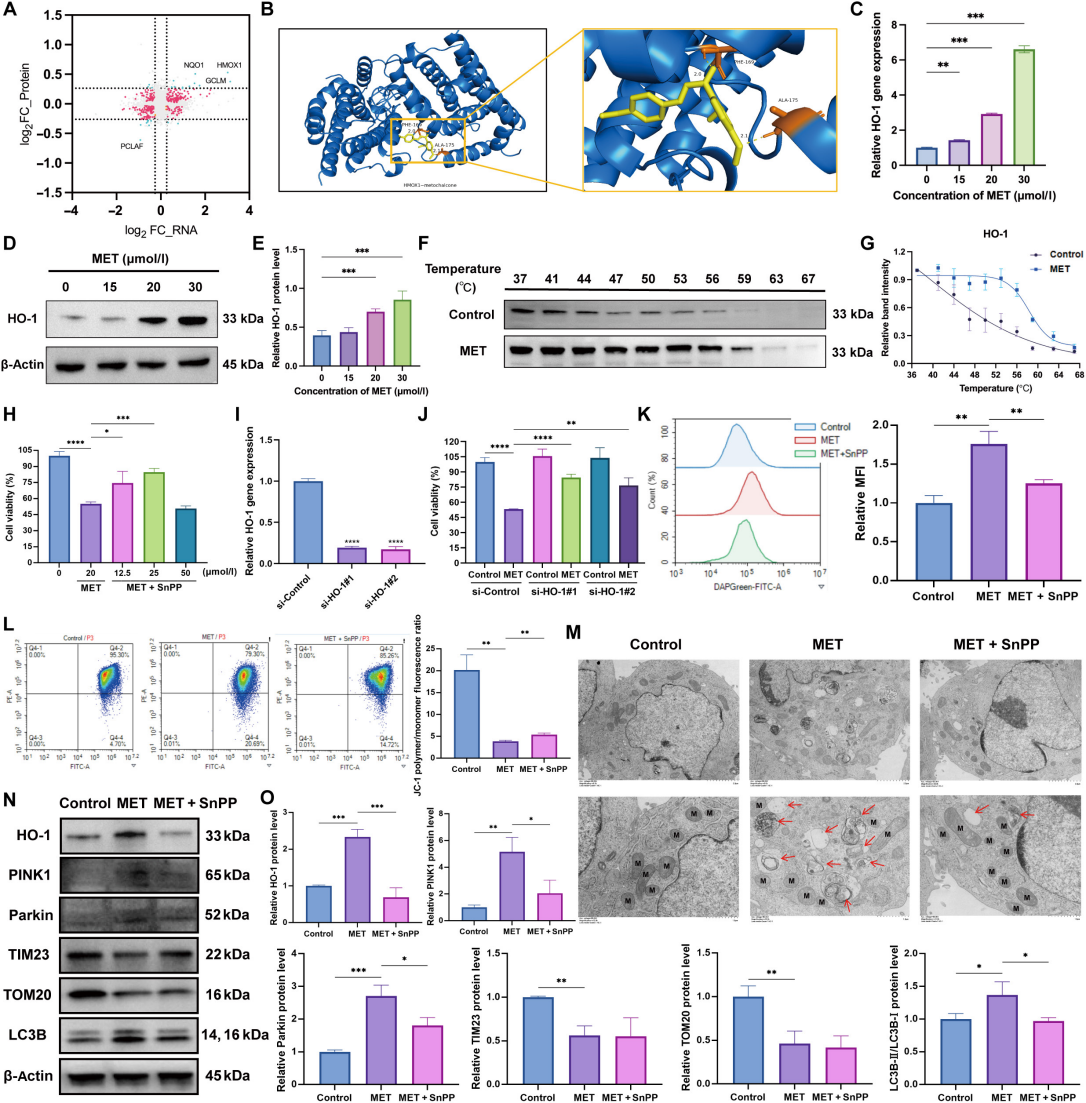
Heme oxygenase-1 (HO-1) serves as a direct target of metochalcone (MET) and participates in mitophagy. (A) Nine-quadrant plot integrating transcriptomic and proteomic fold changes to identify core candidate genes/proteins. HMOX1 (HO-1) showed the most significant up-regulation. (B) Molecular docking diagram of MET with HO-1. (C to E) Quantitative real-time polymerase chain reaction (qRT-PCR) and western blot validation of HO-1 messenger RNA (mRNA) and protein up-regulation upon MET treatment. (F and G) Cellular thermal shift assay (CETSA) showing the thermal stability of HO-1 in the presence and absence of MET. (H) Cell viability of HCT-116 cells after combination treatment of Sn-protopor phyrin (SnPP) and MET. (I) The expression of HO-1 mRNA in HCT-116 cells after si-HO-1 knockdown. (J) Cell viability of HCT-116 cells treated with MET (20 μM) following si-HO-1 knockdown. (K) Detection of autophagy activity by DAPGreen. (L) JC-1 staining for assessment of ΔΨm. (M) Transmission electron microscopy (TEM) images of HCT-116 cells. (N and O) The expression of mitophagy-related proteins in HCT-116 cells after combination treatment of SnPP and MET.

Integrative analysis using a 9-quadrant plot correlated transcriptomic and proteomic fold changes to pinpoint 19 core candidates whose messenger RNA (mRNA) and protein levels changed concordantly (Fig. 4A). Among these, HMOX1 (HO-1) exhibited the most pronounced up-regulation, suggesting an important role in MET-mediated cell death.

To validate HO-1 as a direct MET target, molecular docking and cellular thermal shift assay (CETSA) were performed. Molecular docking showed that MET bound HO-1 with Δ*G* = −6.49 kJ/mol via hydrogen bonds at PHE-169 and ALA-175 (Fig. 4B). Quantitative real-time polymerase chain reaction (PCR) and western blot confirmed significant up-regulation of HO-1 mRNA and protein upon MET treatment (Fig. 4C to E). CETSA revealed that MET increased the melting temperature of HO-1 from 38.8 to 58.3 °C (Fig. 4F and G), enhancing thermal stability.

To further assess the functional role of HO-1, cells were cotreated with the HO-1 inhibitor Sn-protopor phyrin (SnPP) and MET (20 μM). When co-administered with MET, SnPP at 12.5 and 25 μM significantly rescued MET-induced cytotoxicity in HCT-116 cells, whereas this protective effect was lost at 50 μM SnPP (Fig. 4H), likely due to its intrinsic cytotoxicity at higher concentrations (Fig. S1).

Given the potential off-target toxicity of SnPP, we further validated these findings using siRNA-mediated knockdown of HO-1, which achieved efficient suppression of HO-1 expression (Fig. 4I). Following MET treatment, HO-1 silencing considerably attenuated MET-induced cytotoxicity in HCT-116 cells, further confirming that HO-1 contributes to the cytotoxic effect of MET (Fig. 4J).

Subsequently, we cotreated cells with SnPP (25 μM) and MET (20 μM) to examine their effects on mitophagy-related parameters. Compared to MET alone, the addition of SnPP decreased DAPGreen levels and increased mitochondrial membrane potential, suggesting that HO-1 inhibition suppresses MET-induced mitophagy (Fig. 4K and L). Transmission electron microscopy further corroborated these findings: the MET-treated group displayed numerous autophagosomes and autolysosomes (indicated by red arrows in Fig. 4M) along with significant mitochondrial damage. In contrast, the combined MET and SnPP treatment group showed markedly reduced autophagy and relatively normal mitochondrial morphology (Fig. 4M). Western blot analysis further revealed that under HO-1 inhibition, the stimulatory effect of MET on the expression of PINK1, Parkin, and LC3B-II was attenuated, while TIM23 and TOM20 remained largely unaffected (Fig. 4N and O).

### MET suppresses tumor growth in mouse models

**Fig. 5.**
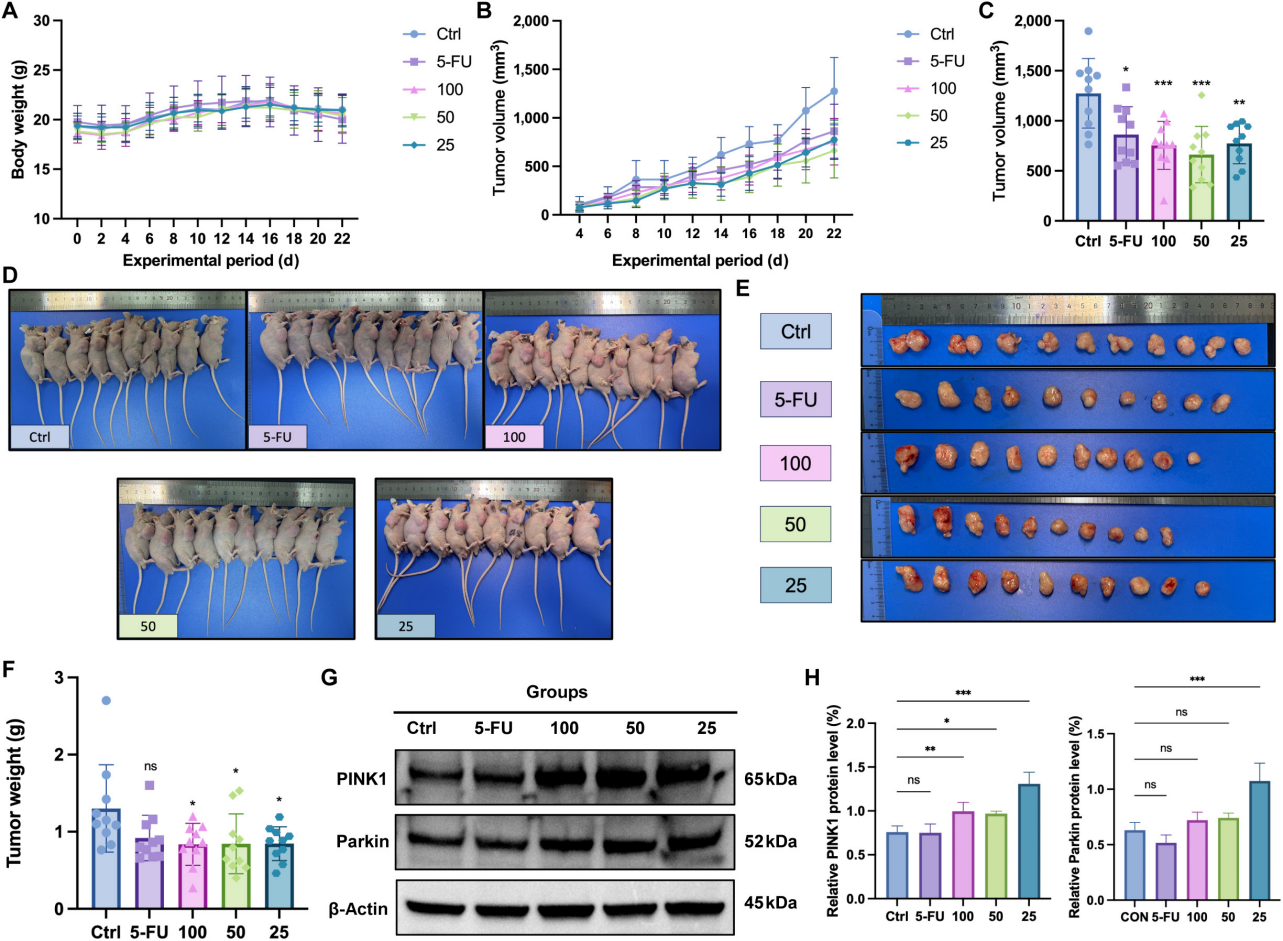
Metochalcone (MET) suppresses tumor growth in HCT-116 xenograft mice. (A) Body weight changes of BALB/c-nu mice treated with different doses of MET or 5-fluorouracil (5-FU). (B to E) Tumor volume measurements and images of mice from each group. (F) Tumor weights at the end of the study. (G and H) Western blot analysis of PINK1 and Parkin protein expression in excised tumor tissues from different treatment groups.

To evaluate the anti-tumor efficacy of MET in vivo, a subcutaneous HCT-116 xenograft model in BALB/c-nu mice was established. Throughout the 21-d dosing regimen, MET-treated mice maintained body weights compared to controls (Fig. 5A), indicating minimal systemic toxicity. The tumor volumes in all MET groups grew markedly more slowly than those in the vehicle group (Fig. 5B and C). At the study’s end, tumor weights were reduced by 35.8%, 35.2%, and 35.1% in high-, medium-, and low-dose MET, respectively (*P* < 0.05 vs. vehicle), whereas 5-FU achieved a 29.5% reduction (*P* = 0.0797) (Fig. 5D to F). Western blot analysis of excised tumors revealed up-regulation of the PINK1 and Parkin proteins in MET-treated mice compared to those in controls (Fig. 5G and H), supporting an in vivo activation of mitophagy.

**Fig. 6.**
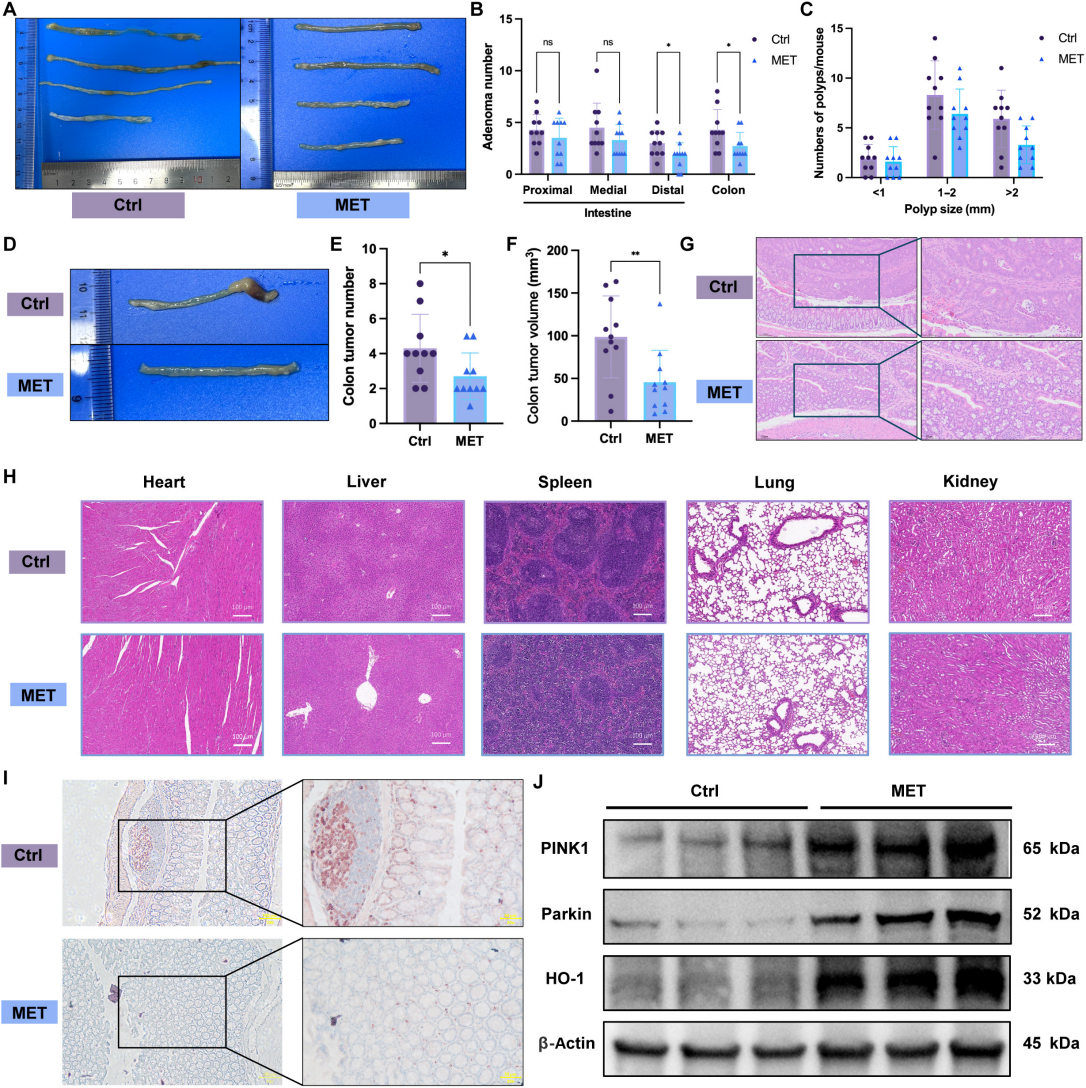
Metochalcone (MET) reduces intestinal adenoma burden in *Apc*^Min/+^ spontaneous colorectal cancer (CRC) mice. (A and B) Representative images and total polyp counts in the small intestine and colon of *Apc*^Min/+^ mice treated with MET or vehicle. (C) Distribution of polyps by diameter. (D to F) Representative images and total polyp number and volume in the colon. (G and H) Representative hematoxylin and eosin (H&E) staining of colon sections and major organs. (I) Ki-67 immunohistochemistry staining and quantitative analysis. (J) Western blot analysis of heme oxygenase-1 (HO-1), PINK1, and Parkin protein expression in tumor tissues.

In the *Apc*^Min/+^ spontaneous CRC model (MET 100 mg/kg vs. vehicle, *n* = 10 per group), MET treatment markedly decreased intestinal adenoma burden. Total polyp counts were substantially lower in MET-treated mice, with the most pronounced reductions in the distal small intestine and colon (*P* < 0.05) (Fig. 6A and B). Analysis by tumor diameter further revealed fewer lesions in both the 1 to 2 and >2 mm categories (Fig. 6C). In the colon specifically, MET considerably reduced both polyp number and volume compared to the vehicle (*P* < 0.05) (Fig. 6D to F). Histopathological examination of colon sections by hematoxylin and eosin (H&E) revealed that vehicle-treated mice exhibited dysplastic tumor nodules with nuclear atypia and inflammatory infiltrates, whereas MET-treated colons retained normal mucosal architecture with only focal inflammation (Fig. 6G). Furthermore, we performed H&E staining on the major organs, including the heart, liver, spleen, lung, and kidney. Compared to the control group, no significant pathological damage was observed in any of these organs from the MET-treated group, indicating a favorable safety profile for MET (Fig. 6H). Ki-67 immunohistochemistry showed a lower proliferation index in MET-treated tumors (Fig. 6I). Consistent with the xenograft findings, MET up-regulated HO-1, PINK1, and Parkin in tumor tissues (Fig. 6J).

Collectively, these data demonstrate that MET potently inhibits CRC tumor growth in both xenograft and genetically driven models, with mechanistic evidence of HO-1 up-regulation and PINK1/Parkin-mediated mitophagy in tumor tissues.

### MET is associated with changes in gut microbiota composition in *Apc*^Min/+^ mice

**Fig. 7.**
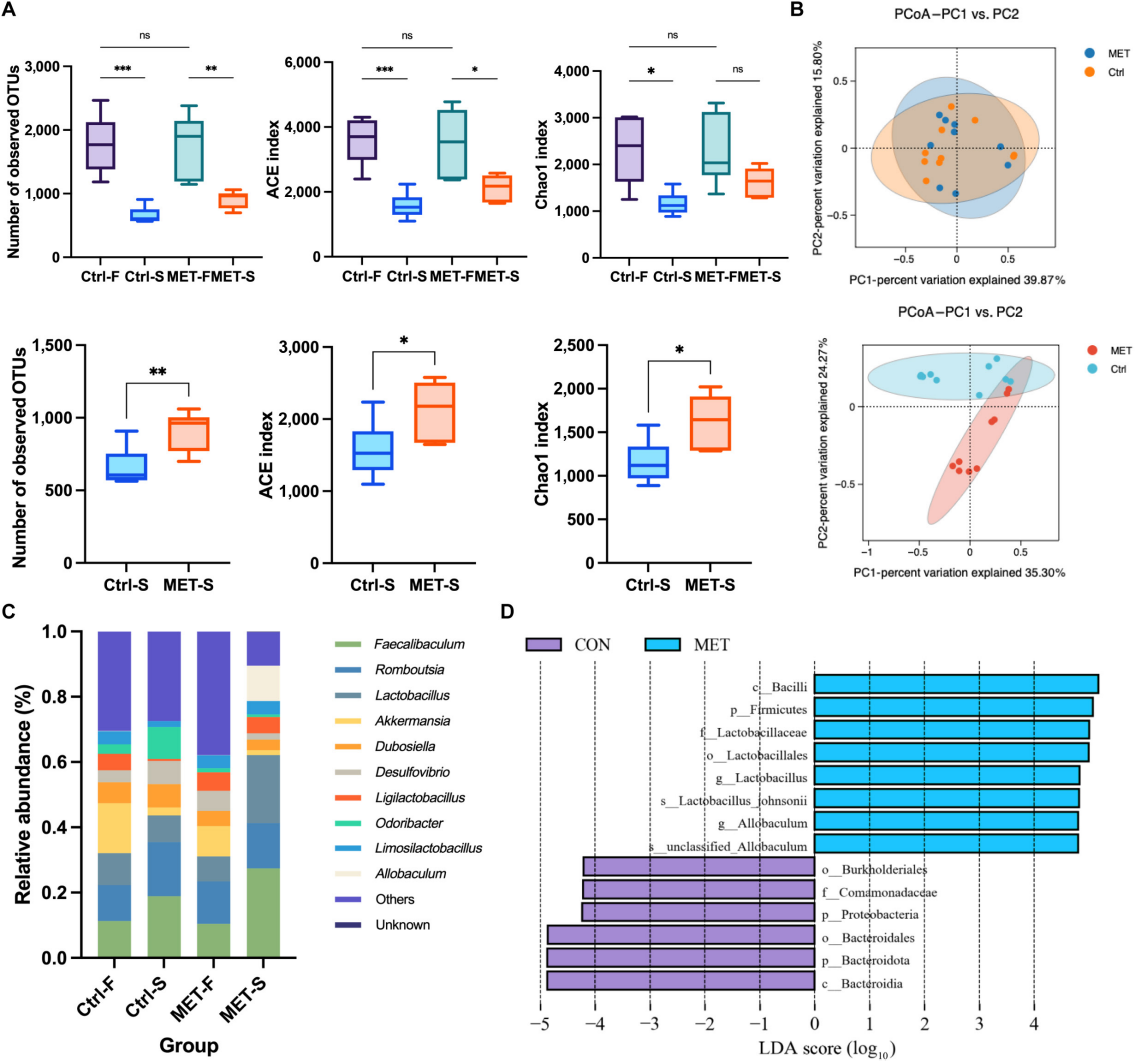
Metochalcone modulates gut microbiota composition in *Apc*^Min/+^ mice. (A) α-diversity indices (operational taxonomic units [OTUs], abundance-based coverage estimator [ACE], and Chao1) of fecal samples at week 0 and week 4. (B) Principal coordinates analysis (PCoA) plot of β-diversity, showing distinct microbial community clustering between groups at week 4. (C) Histogram of species of distribution. (D) Linear discriminant analysis effect size (LEfSe) plot.

To investigate changes in gut microbial composition during MET treatment in colorectal tumorigenesis, fecal samples from *Apc*^Min/+^ mice were collected at week 0 and week 4 for 16S ribosomal RNA (rRNA) gene sequencing. α-diversity indices, including operational taxonomic units, abundance-based coverage estimator, and Chao1, revealed a reduction in microbial richness in both groups at week 4; however, this decline appeared less pronounced in the MET-treated group compared with that in controls (Fig. 7A), suggesting a relative preservation of microbial diversity.

Beta diversity analysis further confirmed shifts in microbial community structure. Principal coordinates analysis revealed similar microbial profiles between groups at baseline, whereas distinct clustering was observed at week 4, indicating time-associated differences in gut microbiota composition in response to MET treatment (Fig. 7B). Taxonomic profiling demonstrated that the relative abundance of *Lactobacillus* increased in the MET-treated group at week 4 (0.209%) compared with that at week 0 (0.076%), whereas *Desulfovibrionaceae* showed a decrease over the same period (Fig. 7C). In contrast, the control group exhibited an opposite trend, with a reduction in *Lactobacillus* and an increase in *Desulfovibrionaceae*.

Given that *Lactobacillus*, particularly *Lactobacillus johnsonii*, has been associated with anti-inflammatory effects and maintenance of intestinal barrier integrity and *Desulfovibrionaceae* has been linked to oxidative stress and inflammation, these microbial shifts may reflect a more favorable gut microbiota profile in MET-treated mice. Linear discriminant analysis effect size analysis identified *Lactobacillus*, especially *L. johnsonii*, as significantly enriched in the MET-treated group (Fig. 7D). These microbiota alterations were observed in association with MET treatment and tumor progression.

## Conclusion

CRC continues to impose a substantial global mortality burden, underscoring the demand for safer and more effective therapeutic options. This study demonstrates that MET, a synthetic chalcone derivative, exerts significant anti-CRC activity through multiple mechanisms, including inhibition of cell proliferation and motility, induction of G_0_/G_1_ cell-cycle arrest, and activation of mitophagy, and is associated with alterations in gut microbiome composition. Considering the increasingly recognized role of regulated cell death (RCD) in shaping the tumor microenvironment and therapeutic outcomes, the ability of MET to simultaneously induce apoptosis and activate specific RCD pathways like mitophagy presents a strong rationale for its future investigation as a novel immunotherapeutic sensitizer [30–33].

Emerging evidence underscores the tumor-suppressive role of PINK1-mediated mitophagy in CRC. Clinical specimens reveal that PINK1 expression is markedly reduced in human colorectal tumors compared with that in adjacent normal mucosa. In support, genetic deletion of PINK1 accelerates colon tumorigenesis in both azoxymethane/dextran sodium sulfate and *Apc*^Min/+^ mouse models [34], highlighting the importance of mitophagy in clearing damaged mitochondria and restraining neoplastic transformation. Moreover, PINK1 loss promoted colon tumorigenesis by increasing mitochondrial iron and oxidative stress. Pharmacologic or genetic reduction of mitochondrial iron and superoxide levels suppressed tumor growth in vitro and in vivo, revealing a therapeutic vulnerability in PINK1-low CRC [35].

HO-1 is a stress-inducible enzyme critical for maintaining cellular homeostasis and mitigating oxidative stress [36]. Most studies indicate that HO-1 is highly expressed in various tumors and promotes their growth. HO-1 can activate the Nrf2 pathway in non-small cell lung cancer cisplatin resistance and gastric cancer metastasis [37,38], inhibit glioblastoma ferroptosis [39], and induce immunosuppression in ovarian cancer [40]. Indeed, its complexity is underscored by instances where it exhibits anti-tumor effects. For example, the antiplatelet drug vorapaxar can up-regulate HO-1 to promote mitochondrial ferroptosis and enhance anti-tumor immunity [41], and ISL also induces gastric cancer cell ferroptosis via HO-1 up-regulation, indicating its tumor-suppressive potential [42].

Leveraging data from The Cancer Genome Atlas Program database, we observed no significant differential expression of HO-1 in colon adenocarcinoma, and while higher expression tended toward better prognosis, this trend was not statistically significant (Fig. S2A and B). In rectal adenocarcinoma (READ), HO-1 was substantially down-regulated, with elevated expression correlating with improved prognosis (Fig. S2C and D). These findings further confirm the complexity of HO-1’s role, particularly as it exhibits distinct patterns across different subtypes of CRC. The association of lower HO-1 expression with poorer prognosis, and, conversely, higher expression with improved outcomes in READ, strongly suggests a potential tumor-suppressive role for HO-1 in this specific cancer type. This observation aligns robustly with our experimental results demonstrating that MET promotes HO-1 expression to inhibit CRC progression. Collectively, our data support HO-1 as a potential tumor suppressor, at least within certain CRC subtypes, rather than a universal pro-oncogenic factor. This provides a crucial foundation for further elucidation of HO-1’s precise mechanisms in CRC and its potential as a therapeutic target.

The intimate connection between HO-1 and mitophagy is increasingly recognized. For instance, *Marsdenia tenacissima* extract induces HO-1 expression and mitochondrial translocation, thereby activating PINK1/Parkin-mediated mitophagy to accelerate ferroptosis and elicit anti-tumor effects in osteosarcoma [43]. Similarly, uric acid activates the Nrf2/HO-1 pathway, stimulating analogous mitophagy to alleviate mitochondrial dysfunction and neuronal apoptosis in Alzheimer’s disease [44]. Leveraging integrated multi-omics and experimental validation, our work herein conclusively identifies HO-1 as a pivotal gene in MET-induced mitophagy within CRC cells.

Natural compounds are actively being investigated for their potential roles in CRC prevention and treatment, partly through associations with gut microbiota composition, including changes in the relative abundance of beneficial and harmful bacterial taxa [45,46]. Our gut microbiome analysis showed that MET treatment was associated with a less pronounced decline in α-diversity in *Apc*^Min/+^ mice and with shifts in community composition characterized by an increased relative abundance of *Lactobacillus* and a decreased abundance of *Desulfovibrionaceae*. Increased abundance of *Lactobacillus* may be associated with the anti-tumor effects observed for MET, potentially through its known roles in supporting intestinal barrier function and regulating local inflammatory responses. Indeed, clinical and preclinical studies have demonstrated that supplementation with *L. johnsonii* following colorectal surgery can reduce pathogenic bacterial loads and modulate mucosal immunity in CRC patients [47], while oral administration of *L. johnsonii* has been shown to enhance CD8+ T-cell infiltration and improve responses to anti-PD-1 immunotherapy [48]. Conversely, overgrowth of *Desulfovibrio* species, sulfate-reducing bacteria enriched in high-fat diet-fed *Apc*^Min/+^ mice, promoted CRC cell proliferation, migration, and invasion [49]. Together, these observations suggest that MET treatment is associated with gut microbiota changes that may reflect a shift toward a composition potentially more favorable for CRC treatment.

Beyond these microbiota alterations, potential interactions between gut microbial changes and mitophagy-related processes should also be considered. For instance, the significant increase in beneficial bacteria like *Lactobacillus* observed after MET treatment could lead to an elevated production of short-chain fatty acids, such as butyrate. Butyrate is a well-established modulator of host cell metabolism and has been shown to directly or indirectly regulate mitochondrial function and induce or enhance mitophagy in various cell types [50,51]. Therefore, it is possible that gut-microbiota-associated metabolic changes may interact with MET-induced mitophagy; however, this relationship remains speculative and requires further functional validation. Overall, these observations highlight a potential interplay between microbial composition and mitochondrial quality control pathways in the context of MET treatment.

While our current study provides strong correlative evidence for these microbial shifts, future investigations employing in vitro microbiota cultivation or germ-free/antibiotic-treated mouse models will be essential to definitively establish the direct causal mechanisms underlying MET’s beneficial effects on the gut microbiome and its subsequent anti-tumor efficacy.

In summary, MET emerges as a promising CRC therapeutic that inhibits proliferation and metastasis, induces G_0_/G_1_ arrest, and triggers mitochondrial dysfunction and mitophagy, accompanied by HO-1 up-regulation and activation of the PINK1/Parkin pathway, while effectively suppressing tumor growth in vivo and is associated with alterations in gut microbiome composition.

## Materials and Methods

### Reagent and cell culture

MET was purchased from WuXi AppTec, China (≥98% purity). A 10 mM stock solution of MET was prepared by dissolving the compound in dimethyl sulfoxide and subsequently stored at −20 °C. For in vitro experiments, MET was diluted in McCoy’s 5A medium containing 1% fetal bovine serum (FBS) (Gibco, USA) to the final concentrations. For in vivo studies, MET was prepared using formation of 2:8:40:50 (v/v/v/v) dimethyl sulfoxide/Tween-80/PEG-300/saline. The same vehicle formulation was used for all control groups.

SnPP was purchased from MCE (China).

HCT-116 cells were obtained from the National Collection of Authenticated Cell Cultures (China). HT-29 and NCM460 cells were bought from Quicell (China). MC38 and SW480 were bought from Pricella (China). HCT-116 and HT-29 cells were maintained in McCoy’s 5A medium enriched with 10% FBS. NCM460 cells were cultured in RPMI 1640 supplemented with 10% FBS. MC38 and SW480 were cultured in Dulbecco’s modified Eagle medium supplemented with 10% FBS. They were placed at 37 °C in a humidified chamber with 5% CO_2_.

### Cell Counting Kit-8 assay

Cells were plated into 96-well plates (5 × 10^3^ cells /well) and exposed to drugs in different concentrations for 24 h. The Cell Counting Kit-8 reagent (DOJINDO, Japan) was then added to each well containing fresh medium, and the absorbance at 450 nm was measured after incubation.

### Transcriptomic and proteomic analysis

HCT-116 cells in the logarithmic growth phase were exposed to MET (15 μM) or vehicle control for 24 h, harvested by trypsinization, washed twice with phosphate-buffered saline (PBS), and pelleted. Total RNA was submitted to BMKCloud (China) for paired-end sequencing (Illumina NovaSeq 6000, PE150, ~6.1 Gb/sample).

Total protein was extracted in radioimmunoprecipitation assay (RIPA) buffer, quantified by bicinchoninic acid assay, and samples were submitted to Oebiotech (China) for tandem mass tag labeling and liquid chromatography–tandem mass spectrometry analysis. DEGs (|FC| ≥ 2, false discovery rate < 0.01) were identified by DESeq2, and DEPs (|FC| ≥ 1.2, *P* < 0.05) were determined. GO and KEGG enrichment analyses were performed for DEGs and DEPs. Integration of transcriptomic and proteomic fold changes via 9-quadrant plots highlighted key molecules showing concordant regulation at both mRNA and protein levels, from which mitophagy-related candidates were selected for further validation.

### Molecular docking

The crystal structure of HO-1 (Protein Data Bank [PDB] ID: 1S13; resolution 2.29 Å) was obtained from RCSB PDB. The 3-dimensional structure of MET (PubChem CID: 6063342) was prepared using Open Babel v3.1.1. Docking simulations employed AutoDock v1.5.7. The conformation with the lowest binding energy (Δ*G* = −6.49 kcal/mol) was selected for visualization in PyMOL v2.5.

### Cellular thermal shift assay

HCT-116 cells were seeded into 10-cm dishes (5 × 10^6^ cells/dish) and treated with MET (0 or 15 μmol/l) for 24 h. After trypsinization and PBS washing, cells pellets were resuspended in PBS supplemented with protease inhibitors. Cell suspensions were divided and transferred into 10 tubes and heated for 3 min at temperatures of 37, 41, 44, 47, 50, 53, 56, 59, 63, and 67 °C, followed by incubation at room temperature for 3 min. Following lysis and clarification, soluble fractions were prepared for western blot analysis. Band intensities were quantified using ImageJ, with the 37 °C condition normalized to 100%. Apparent melting curves were fitted using the Boltzmann sigmoid function in GraphPad Prism 10.2.0, and the melting temperature (*T*_m_) was calculated accordingly.

### Transmission electron microscopy

To visualize ultrastructural changes, HCT-116 cells were fixed with glutaraldehyde and osmium tetroxide, dehydrated, resin-embedded, and sectioned for transmission electron microscopy. Ultrathin sections (60 to 80 nm) were prepared and imaged using a Hitachi HT7800 microscope (Hitachi, Japan).

### Detection of intracellular ROS levels

Intracellular ROS levels were assessed using 2′,7′-dichlorodihydrofluorescein diacetate (DOJINDO). HCT-116 cells were cultured under standard conditions for 24 h, loaded with the fluorescent probe and subsequently treated with drugs for 24 h. Cells were washed twice with Hank’s balanced salt solution (HBSS) and incubated with ROS detection working solution for 30 min at 37 °C and then treated with for 24 h, washed with HBSS, trypsinized, and resuspended. Fluorescence intensity was analyzed by flow cytometry using the fluorescein isothiocyanate channel to quantify intracellular ROS levels.

### Mitochondrial superoxide and membrane potential assays

Mitochondrial superoxide levels were evaluated using the MitoSOX Red mitochondrial superoxide indicator (Invitrogen, USA), while mitochondrial membrane potential (ΔΨm) was determined with the JC-1 Assay Kit (Beyotime) in accordance with the manufacturer’s protocol. HCT-116 cells were treated as indicated and analyzed by flow cytometry; mean MitoSOX fluorescence intensity served as the readout for mitochondrial superoxide production and the red/green fluorescence ratio calculated to indicate ΔΨm.

### Mitophagy assay

To evaluate mitophagic activity, HCT-116 cells were subjected to dual-fluorescence labeling using the Mitophagy Detection Kit (DOJINDO), containing the Mtphagy dye and Lyso dye. HCT-116 cells were seeded into 35-mm confocal dishes (1 × 10^5^ cells/dish) and incubated for 24 h, then washed twice with HBSS, and stained with 100 nmol/l Mtphagy dye working solution at 37 °C for 30 min. After 2 HBSS washes, cells were treated with MET (0 or 15 μM) for 24 h, washed again, and counterstained with Hoechst 33342 (Beyotime) at 37 °C for 10 min. Following 2 additional washes, the Lyso dye (1 μM) was applied for 30 min at 37 °C. Cells were washed twice with HBSS, and the fluorescence images were captured using a Zeiss LSM 880 confocal laser scanning microscope.

### Cell transfection

HCT-116 cells were inoculated into 6-well plates and cultured for 24 h. At a cell density of 60% to 70%, the cells were transfected with 2.5 μg of siRNA against PINK1 or HO-1, and the other group used the same amount of siRNA as a negative control. The siRNA was synthesized by GenePharma (China) with transfection efficiency >80%.

### Western blot analysis

Total protein extraction from HCT-116 cells was performed using RIPA lysis, and the mitochondrial protein was extracted using a mitochondrial isolation and protein extraction kit (Proteintech). Protein samples were separated by sodium dodecyl sulfate–polyacrylamide gel electrophoresis, transferred to polyvinylidene fluoride membranes, probed with primary and horseradish peroxidase (HRP)-conjugated secondary antibodies, and visualized by chemiluminescence. Densitometry was performed using ImageJ (version 1.54g). For tissue samples, fresh specimens were snap-frozen, homogenized in ice-cold RIPA with protease inhibitors, centrifuged, and supernatants processed as above.

### Quantitative real-time PCR

Total RNA was isolated with TRIzol and reverse-transcribed using the PrimeScript RT reagent kit with gDNA Eraser (TAKARA, Japan). Quantitative PCR was performed with the PowerUp SYBR Green Master Mix (Thermo Fisher Scientific, USA) on a QuantStudio 3 system under standard cycling conditions.

The primer sequences were as follows: for HO-1, 5′-AAGACTGCGTTCCTGCTCAAC-3′ and 5′-AAAGCCCTACAGCAACTGTCG-3′; for β-actin, 5′-CTCCATCCTGGCCTCGCTGT-3′ and 5′-GCTGTCACCTTCACCGTTCC-3′.

### Mouse model

Four-week-old male BALB/c-nu mice were purchased from Chengdu Dossy Experimental Animal Co. and acclimatized for 1 week under specific-pathogen-free conditions (22 ± 2 °C, 50% ± 10% humidity, 12-h light/dark cycle). HCT-116 cells (1 × 10^7^ cells/ml) were suspended in ice-cold PBS/Matrigel (1:1 v/v), and 0.2 ml was injected subcutaneously into the right flank. Mice with confirmed tumor formation at day 7 postinoculation were randomized into 5 groups (*n* = 10/group): (a) vehicle control, (b) positive control (5-FU, 25 mg/kg; MedChemExpress, USA), and (c to e) MET (25, 50, or 100 mg/kg). Intraperitoneal injections were administered every other day for 21 d. Tumor diameters were measured every 48 h using digital calipers, with the volume calculated as *V* = (*L* × *W*^2^)/2 (*L*, longest axis; *W*, shortest axis). Body weight and survival were monitored throughout the study.

Six-week-old male *Apc*^Min/+^ mice were purchased from GemPharmatech Co., Ltd and acclimatized for 1 week. Mice were randomly divided into 2 groups (*n* = 10/group): control and MET (100 mg/kg) by oral gavage every other day, concurrently maintained on a 60% high-fat diet (Research Diets, USA) for 8 weeks. General health, body weight, activity, and fecal appearance were monitored. At the end of the experiment, mice were fasted for 12 h and euthanized, and the entire intestine was harvested. The intestines were flushed with ice-cold PBS, longitudinally opened, and divided into proximal, middle, and distal sections. Adenomas were assessed under a stereomicroscope and classified by size (>2, 1 to 2, and <1 mm).

All animal procedures were approved by the Institutional Animal Care and Use Committee of Sichuan University (Approval No. K2025016) and conducted in accordance with institutional guidelines.

### 16S rRNA gene sequencing and microbiome analysis

Fresh feces of *Apc*^Min/+^ mice were collected for 16S rRNA sequencing. Bacterial 16S rRNA genes were amplified using barcoded primers 27F (AGRGTTTGATYNTGGCTCAG) and 1492R (TASGGHTACCTTGTTASGACTT), and the resulting amplicons were purified, quantified, and pooled in equimolar amounts. SMRTbell libraries were constructed and sequenced on a PacBio Sequel II platform. Sequencing data were processed and analyzed using the BMKCloud platform, including taxonomic assignment based on the SILVA 138.1 database and downstream microbial community analyses.

### Histology and immunohistochemistry staining

Collected tissues were fixed and processed for paraffin embedding, after which serial sections (5 μm) were prepared for histological evaluation. H&E staining was performed using routine procedures. For immunohistochemical analysis, intestinal sections from *Apc*^Min/+^ mice were processed using standard procedures, including deparaffinization, antigen retrieval, blocking, incubation with Ki-67 primary antibody (1:200; Proteintech, China), and detection with HRP-conjugated secondary antibody and DAB chromogen.

### Statistical analysis

Data are expressed as mean ± standard error of the mean. Statistical analysis was performed with a one-way analysis of variance with a Tukey test for multiple comparisons. Two-group comparisons were made using a Student *t* test. All analyses were performed using Prism 10.2.0 (GraphPad). The value of *P* < 0.05 was considered significant. Statistical significance is indicated as *P < 0.05, **P < 0.01, ***P < 0.001, and ****P < 0.0001 compared with the control group.

## Data Availability

The datasets during the current study are available from the corresponding authors on reasonable request.
