# Supplementary material for: HO-1 Up-regulation and PINK1/Parkin-Mediated Mitophagy Contribute to the Anti-tumor Effects of Metochalcone in Colorectal Cancer
Source: Research (Wash D C). 2026 Aug 4;9:1387. doi: 10.34133/research.1387 (PMC13433928; doi:10.34133/research.1387)
Supplement: Supplementary file 1 [file research.1387.f1.docx]

**Fig. S1. Effects of SnPP on cell viability of HCT-116 cells.**

**Fig. S2. Differential expression and prognostic significance of HO-1 in CRC based on TCGA data.** (A) HO-1 expression in colon adenocarcinoma (COAD) tissues compared to normal tissues. (B) Kaplan-Meier curve showing the overall survival of COAD patients stratified by HO-1 expression levels. (C) HO-1 expression in rectal adenocarcinoma (READ) tissues compared to normal tissues. (D) Kaplan-Meier curve showing the overall survival of READ patients stratified by HO-1 expression levels.
